# Polar Auxin Transport Determines Adventitious Root Emergence and Growth in Rice

**DOI:** 10.3389/fpls.2019.00444

**Published:** 2019-04-09

**Authors:** Chen Lin, Margret Sauter

**Affiliations:** Plant Developmental Biology and Physiology, University of Kiel, Kiel, Germany

**Keywords:** adventitious roots, polar auxin transport, auxin efflux carrier, root angle, root emergence, rice, flooding

## Abstract

Flooding is a severe limitation for crop production worldwide. Unlike other crop plants, rice (*Oryza sativa* L.) is well adapted to partial submergence rendering it a suitable crop plant to understand flooding tolerance. Formation of adventitious roots (ARs), that support or replace the main root system, is a characteristic response to flooding. In rice, AR emergence is induced by ethylene and in the dark where roots grow upward. We used the synthetic auxins 2,4-D and α-NAA, and the auxin transport inhibitor naphthylphtalamic acid (NPA) to study emergence, growth rate and growth angle of ARs. While α-NAA had no effect, NPA and 2,4-D reduced the root elongation rate and the angle with a stronger effect on root angle in the dark than in the light. Furthermore, NPA delayed emergence of AR primordia suggesting that efflux carrier-mediated auxin transport is required for all aspects of directed AR growth. Expression analysis using *OsPIN:GUS* reporter lines revealed that *OsPIN1b* and *OsPIN1c* promoters were active in the stele and root cap in accord with their predicted role in acropetal auxin transport. *OsPIN2* was expressed at the root tip and was reduced in the presence of NPA. Auxin activity, detected with *DR5:VENUS*, increased in primordia following growth induction. By contrast, auxin activity was high in epidermal cells above primordia and declined following growth induction suggesting that auxin levels are antagonistically regulated in AR primordia and in epidermal cells above AR primordia suggesting that auxin signaling contributes to the coordinated processes of epidermal cell death and AR emergence.

## Introduction

Rice is semi-aquatic showing remarkable tolerance to partial submergence. Adventitious root (AR) primordia form at each node during normal development that emerge during submergence to replace the original dysfunctional root system (Sauter, 2013). AR formation is regarded as a characteristic response to flooding not only in rice but also in other flooding tolerant and flooding-intolerant plants such as tomato (*Lycopersicon esculentum*) (Vidoz et al., 2010) and pecan tree (*Catya illinoinensis*) (Smith and Ager, 1988). ARs are located closer to the shoot than the original root system thereby facilitating oxygen supply. Furthermore, ARs take up oxygen from flood waters further improving their oxygen status. Recent work on rice revealed that ARs grow upward in the dark such that roots get closer to the oxygen-rich water surface (Lin and Sauter, 2018). The hormones and molecular mechanisms that control AR growth direction need yet to be elucidated. This study sets out with the mechanistic analysis by studying the role of auxin transport and signaling in AR growth. In the past decades, the number of flooding events has increased worldwide causing increasing crop losses (Voesenek and Bailey-Serres, 2015). It is hence rather timely to decipher in more depth the molecular mechanisms that control AR growth in flooded crop plants.

In rice and *Solanum dulcamara*, the gaseous hormone ethylene that accumulates in submerged tissues due to a reduced diffusion rate in water, promotes AR growth (Drew and Sisworo, 1979; Lorbiecke and Sauter, 1999; Dawood et al., 2016). Both, ethylene-induced AR emergence and AR growth rate are enhanced by gibberellic acid and inhibited by abscisic acid (Steffens and Sauter, 2005; Steffens et al., 2006).

Unlike growth of nodal ARs, growth of crown roots in rice seedlings is inhibited by ethylene. Inhibition of root growth by ethylene and auxin has been reported for Arabidopsis where ethylene and auxin pathways interact (Stepanova et al., 2007; Swarup et al., 2007). A recent study in rice seedlings revealed that auxin signaling downstream of ethylene is required for root growth inhibition by ethylene (Chen et al., 2018) suggesting similar hormonal crosstalk in monocots and dicots. Auxin signal transduction through SCF^TIR1/AFBs^-Aux/IAAs has been well studied (Salehin et al., 2015). The E3 ubiquitin ligase SCF^TIR1/AFB^ recognizes Aux/IAA proteins as a target upon binding of auxin to the F-Box subunit TIR1/AFB resulting in their ubiquitination and proteasome-dependent degradation. Canonical Aux/IAA proteins act as transcriptional repressors of auxin response factors (ARFs). Their degradation allows transcription of ARFs which regulate auxin-responsive genes including most Aux/IAA genes, hence providing a negative feedback loop (Salehin et al., 2015). In rice seedlings, ethylene signaling induces *OsIAA26* expression whereby the OsIAA26 protein stability is controlled by the auxin-dependent E3 ubiquitin ligase Soil-surface Rooting 1 (SOR1). This study hence revealed a molecular link between ethylene and auxin signaling in rice root development.

A widely used tool to monitor auxin distribution *in planta* is DR5-based auxin-inducible reporters. The DR5 promoter contains several ARF binding sites resulting in reporter gene induction in the presence of auxin (Ulmasov et al., 1997). A more recently developed auxin reporter uses the destabilizing DII-domain of an Aux/IAA protein that is fused to the fluorescent VENUS protein. An increase in auxin results in degradation of DII-VENUS as an immediate response to SCF^TIR1/AFB^ activation through auxin binding (Brunoud et al., 2012). Local auxin accumulation due to synthesis or polar auxin transport can be tracked with these reporters (Brumos et al., 2018) which aids in deciphering the contribution of an auxin gradient to a development process. Chemiosmosis is widely accepted as mechanism of polar auxin transport (Rubery and Sheldrake, 1974; Raven, 1975; Adamowski and Friml, 2015). While auxin diffuses passively into the cell through the membrane or by way of facilitated diffusion through AUX1/LAX influx carriers, the efflux of the IAA anion requires auxin efflux carriers of the PIN-FORMED (PIN) protein family or ATP-Binding Cassette family B (ABCB) transporters. PIN and ABCB activity is a target of the auxin transport inhibitor 1-naphthylphtalamic acid (NPA) and of flavonoids that interfere with the interaction of ABCB and its partner TWISTED DWARF 1 (TWD1) (Bailly et al., 2008). The direction of auxin flux and the establishment of an auxin gradient are achieved by the polar distribution of PINs in the plasma membrane (Tanaka et al., 2006). For Arabidopsis, seven PIN genes and for rice, twelve PIN genes were reported (Wang et al., 2009). In Arabidopsis roots, auxin moves acropetally in the central cylinder driven by AtPIN1 activity. Auxin is laterally deflected in the root cap mediated by AtPIN3, AtPIN4 and AtPIN7 activity and moves basipetally in the peripheral cell layers due to AtPIN2 localized in the apical part of the plasma membrane (Vanneste and Friml, 2009). The PIN1 clade has diversified into four members in rice, OsPIN1a-d. OsPIN10a and OsPIN10b are most closely related to AtPIN3, AtPIN4 and AtPIN7, whereas the PIN2 clade is represented by a single member in Arabidopsis and rice (Supplementary Figure S1).

In rice, little is known about the role of auxin and polar auxin transport in the development of ARs. A number of genes related to crown root formation and growth have been identified (Liu et al., 2005; Zhao et al., 2009, 2015). *Crown-rootless 4 (crl4)* and *OsGNOM1* show reduced crown root initiation and delayed lateral root differentiation (Kitomi et al., 2008; Wang et al., 2011). OsGNOM1 controls PIN1 trafficking within the cell, and thus alters polar auxin transport that is required for the formation of an auxin gradient which triggers the asymmetrical division of parenchyma cells that develop into crown roots (Richter et al., 2010). Interestingly, an RNAi-knockdown line for the *OsPIN1b* gene displays a reduced AR penetration rate at the seedling stage (Xu et al., 2005).

In this study, we investigated auxin activity and *OsPIN* gene promoter activities with high spatial resolution during AR emergence and growth. Our results indicate that local auxin gradients determine AR penetration, growth rate and growth direction and thereby have a deep impact on the overall architecture of the AR system.

## Results

### Inhibition of Auxin Transport Inhibits AR Penetration and Growth and Reduces the Root Growth Angle

**FIGURE 1:**
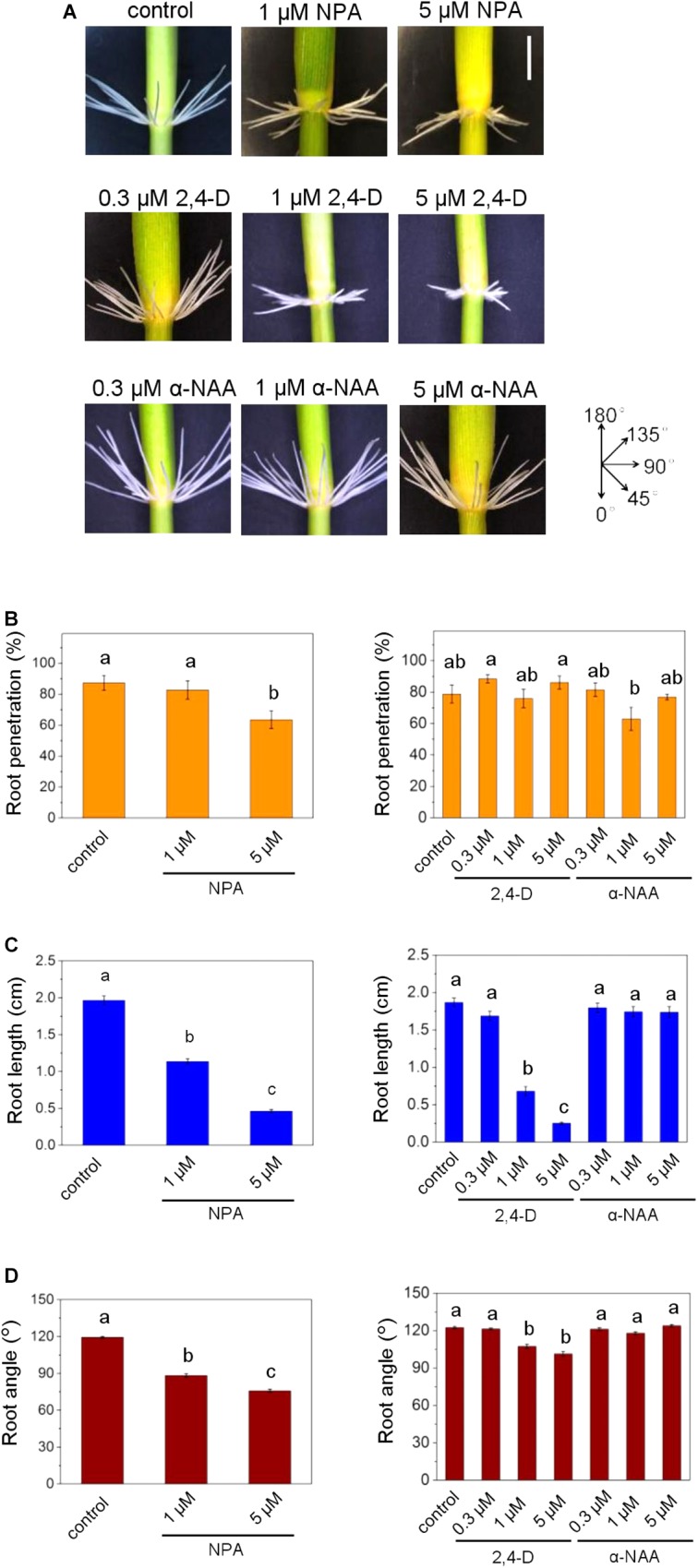
Auxin transport inhibition alters emergence, growth rate and angle of adventitious roots (ARs) in rice. **(A)** ARs were analyzed at the third node using stem sections kept in the dark for 3 days in the presence of NPA, 2,4-D or α-NAA at the concentrations indicated. Phenotypes of ARs exposed to the auxins 2,4-D or α-NAA or to the auxin transporter inhibitor NPA; bar=1 cm. **(B)** Percentage of penetrated ARs. **(C)** Average lengths of penetrated ARs. **(D)** Mean growth angles of ARs. Bars in **B–D** indicate means (±SE) of ARs measured from nine stems per treatment in three independent experiments. Different letters indicate statistically significant differences (*P* < 0.05; ANOVA with Tukey test).

To study the role of auxin on AR growth in rice, we employed the auxin transport inhibitor naphthylphtalamic acid (NPA) and two auxin analogs, 2,4-dichlorophenoxyacetic acid (2,4-D) and 1-naphthalene acetic acid (α-NAA). Rice stem sections were treated with NPA, 2,4-D or α-NAA in the dark (Figure 1). Darkness induced AR emergence with an upward direction of AR growth as described previously (Lin and Sauter, 2018). The root penetration rate, i.e., the number of AR primordia that emerged from the node in relation to the total number of AR primordia, given as percentage, was not significantly altered by 2,4-D or α-NAA at concentrations of 0.3 to 5 μM whereas inhibition of auxin efflux at 5 μM NPA significantly reduced AR penetration (Figure 1B). The length of penetrated ARs was reduced by NPA and 2,4-D in a dose-dependent manner, with an average of 0.3 cm at 5 μM 2,4-D compared to 1.9 cm in the absence of 2,4-D. By contrast, α-NAA did not inhibit AR elongation revealing a functional similarity between auxin efflux inhibition by NPA and 2,4-D activity and a functional difference between the two synthetic auxins 2,4-D and α-NAA (Figure 1C). Similarly, the AR angle decreased in the presence of 2,4-D and NPA but not after exposure to α-NAA (Figure 1D). Taken together, our data suggested that increased intracellular auxin levels induced by chemical inhibition of auxin efflux through NPA or by supplying 2,4-D, that is taken up by cells but not actively secreted by auxin efflux carriers, reduced AR elongation and growth angle whereas the auxin analog α-NAA that is actively exported from the cell through efflux carriers (Delbarre et al., 1996; Simon et al., 2013) did not.

**FIGURE 2:**
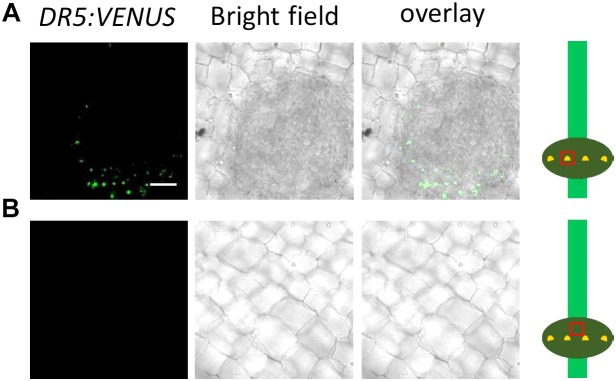
Auxin activity is detected in cells above root primordia but not in other cells. **(A)** Top view on the epidermis and an underlying root primordium at the third node of a *DR5:VENUS* reporter line. Bar ( =25 (um. Green channel: VENUS. **(B)** Top view on the epidermis not covering a root primordium.

Adventitious root emergence is facilitated by programmed death of epidermal cells covering root primordia (Mergemann and Sauter, 2000). To study a possible involvement of auxin in epidermal cell death, we employed a *DR5:VENUS* auxin reporter line (Figure 2A,B). Fluorescence microscopy revealed auxin activity in epidermal cells above root primordia and/or the primordia but not in other epidermal cells indicating a specific function of auxin.

**FIGURE 3:**
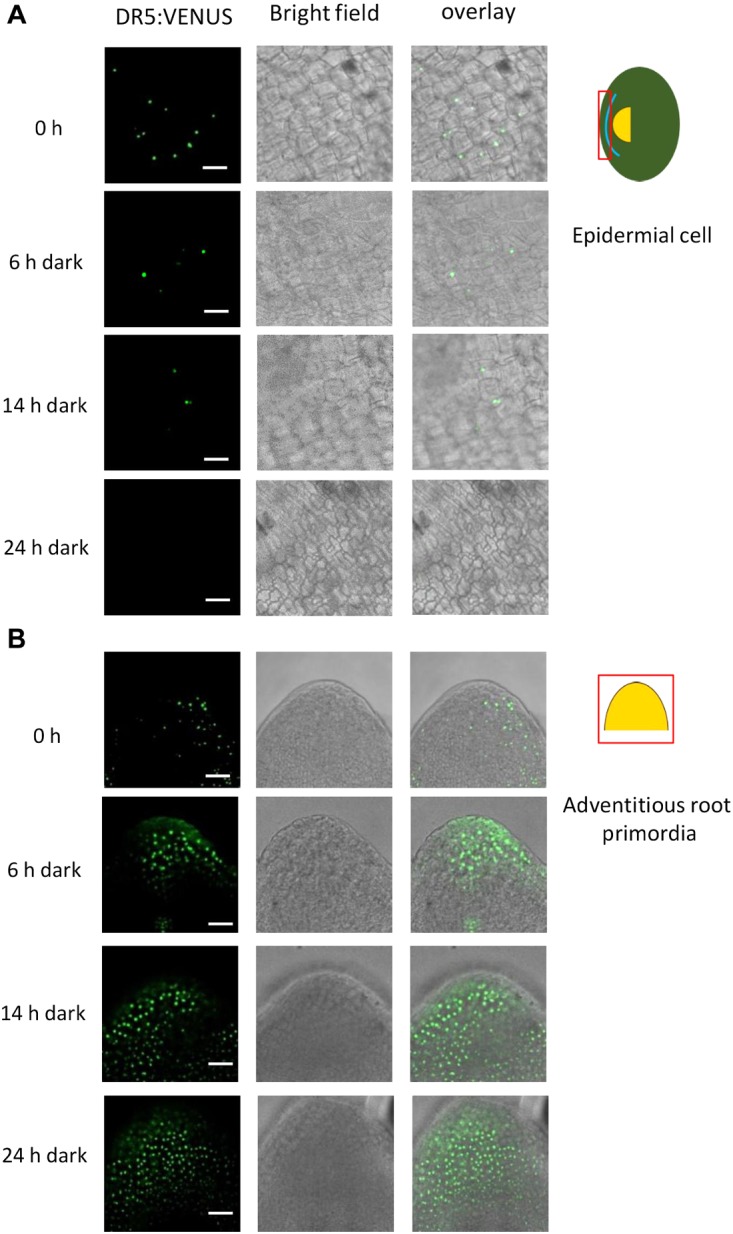
Auxin activity decreases in the epidermis above primordia and increases in AR primordia after induction of AR growth in the dark. Bar ( =25 μm. **(A)** Epidermal peels above a root primordium. Auxin activity was detected by CLSM using the *DR5:VENUS* reporter. **(B)** Isolated AR primordia from the *DR5:VENUS* reporter line.

**FIGURE 4:**
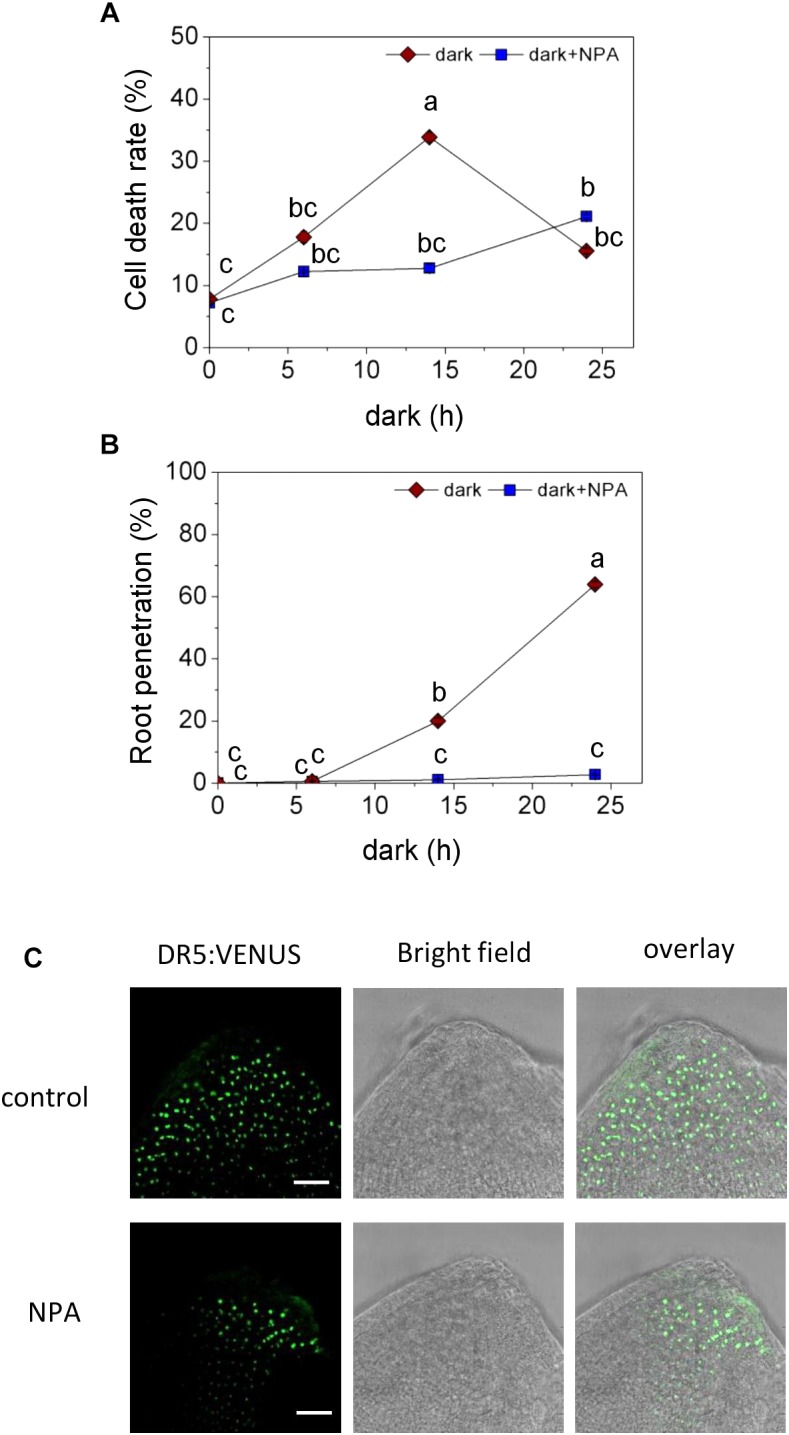
Effect of auxin transport inhibition on programmed cell death (PCD) and AR emergence. **(A)** Cell death of epidermal cells above AR primordia, analyzed with Evans Blue at the third node of stem sections kept in the dark for 6 h, 14 h or 24 h with or without 5 μM NPA. **(B)** Percentage of penetrated ARs. Bars in **A, B** indicate means ( ± SE) of ARs from 9 stems per treatment measured in 3 independent experiments. Different letters indicate statistically significant differences (*P* < 0.05; ANOVA with Tukey test). **(C)** Auxin activity detected with *DR5:VENUS* in AR primordia with or without 5 μM NPA for 1 day; Bar=25 μm.

For better spatial resolution, we peeled off the epidermis above a root primordium and isolated root primordia (Figure 3). Interestingly, when AR growth was induced by transfer to the dark, auxin activity appeared to decline within 6 h in epidermal cells covering a primordium (Figure 3A). At the same time, auxin activity increased in AR primordia following growth induction (Figure 3B). A time course analysis of epidermal cell death (Figure 4A) and root penetration (Figure 4B) showed that 5 μM NPA delayed both and resulted in reduced auxin activity at the AR apex (Figure 4C). These findings suggest that auxin activity coordinates epidermal cell death and AR emergence. The data further indicated that polar auxin transport through efflux carriers controls auxin levels.

### *OsPIN* Genes Are Differentially Expressed in ARs and in Epidermal Cells Above ARs

PINs are carrier proteins that facilitate auxin efflux from plant cells and are responsible for directed auxin flux in plant organs. PINs are encoded by gene families with 7 members in Arabidopsis and 12 members in rice (Supplementary Figure S1) and have mostly been studied in Arabidopsis (Křeček et al., 2009). In Arabidopsis roots, PIN1 drives acropetal auxin transport in the central cylinder, PIN3, 4, and 7 redirect the auxin flux in the root tip to the outer cell layers and PIN2 drives basipetal auxin flow in the outer root cell layers. To find out whether homologous transporters in rice might be involved in generating auxin gradients in ARs and at the site of AR emergence, we obtained GUS reporter lines for the Arabidopsis *PIN1* orthologs *OsPIN1b* and *OsPIN1c*, for the *PIN2* ortholog *OsPIN2* and for *OsPIN10a* which is phylogenetically most closely related to PINs 3, 4 and 7 (Supplementary Figure S1).

**FIGURE 5:**
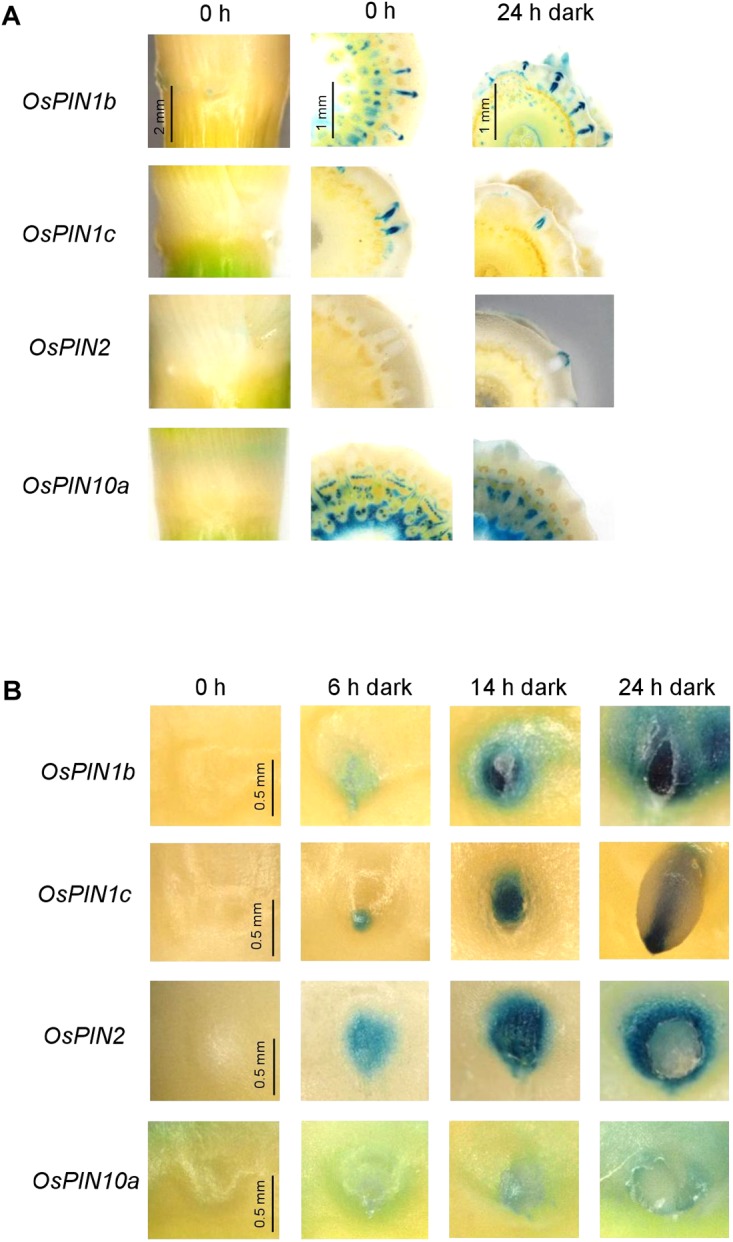
*OsPIN2* expression is induced in epidermal cells above AR primordia after AR growth induction in the dark. **(A)** Expression of *OsPIN1b:GUS, OsPIN1c:GUS, OsPIN2:GUS*, and *OsPIN10a:GUS* was visualized in nodal cross sections following dark exposure for the times indicated. **(B)** Top view on emerging ARs in *OsPIN1b:GUS, OsPIN1c:GUS, OsPIN2:GUS*, and *OsPIN10a:GUS* reporter lines reveals induction of *OsPIN2* expression in the epidermis.

*OsPIN1b:GUS* and *OsPIN1c:GUS* was expressed in AR primordia and penetrated ARs (Figure 5A and Supplementary Figure S2A). *OsPIN2:GUS* expression was not detected in uninduced nodes and appeared in epidermal cells above AR primordia within 6 h after transferring to the dark to trigger AR emergence (Figure 5A,B). *OsPIN2:GUS* was also active at the apex of emerged ARs in what appeared to be the root cap (Supplementary Figure S2). *OsPIN10a:GUS* expression was observed at AR tips within 24 h after transfer to the dark and was maintained during subsequent AR elongation (Figure 5A and Supplementary Figure S2A). Overall, the results revealed regulation of *OsPIN* gene expression following growth-inducing dark treatment. Interestingly, *OsPIN2* expression was induced specifically in epidermal cells above AR primordia following growth induction coincident with reduced auxin activity in these cells (Figure 3A). These findings suggest that OsPIN2-mediated auxin depletion in the epidermis promotes epidermal cell death and root penetration.

### Auxin Transport Is Required for AR Growth Promotion by Ethylene

**FIGURE 6:**
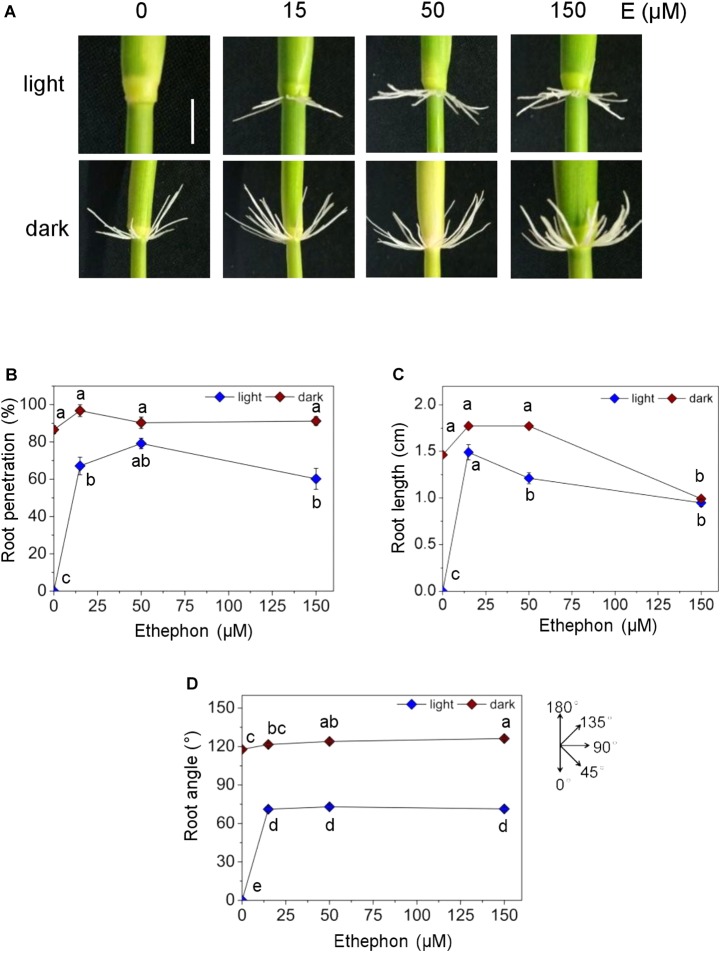
Effect of ethephon on penetration, growth and angle of ARs in the dark. Rice stem sections including the third node were exposed to white light (WL) or kept in the dark for 3 days in the presence of different concentrations of ethephon. **(A**) Phenotypes of AR growth in the dark and in light (bar=1 cm). **(B)** Percentage of root penetration determined with Evans Blue staining. **(C)** Average length of AR. **(D)** Angle of AR growth. Bars in **B–D** indicate means (±SE) from nine stem sections analyzed per treatment in three independent experiments. Different letters indicate statistically significant differences (*P* <0.05; ANOVA with Tukey test).

Adventitious roots emerge in the dark but not in the light (Figure 6A,B). Emergence in the dark was related to dark-induced ethylene synthesis (Fukao et al., 2012) which is in accord with the observation that ethylene promotes emergence and growth of ARs in the light (Figure 6A,B; Mergemann and Sauter, 2000). A dose-response analysis of AR growth revealed that root emergence was elevated to 67.1% at 15 μM ethephon, an ethylene-releasing compound, and remained at this level at higher concentrations while the emergence rate in the dark was close to 100% (Figure 6B). AR elongation was similar in the light and in the dark at 15 and 150 μM ethephon and was lower in the light than in the dark at 50 μM ethephon possibly indicating different responsiveness to ethylene (Figure 6C). ARs grew upward in the dark and downward in the light as described previously (Figure 6A,D; Lin and Sauter, 2018). While the growth angle in the light was independent of ethephon, the growth angle in the dark increased slightly but significantly from 117.8 to 126.2° with increasing ethephon concentration.

**FIGURE 7:**
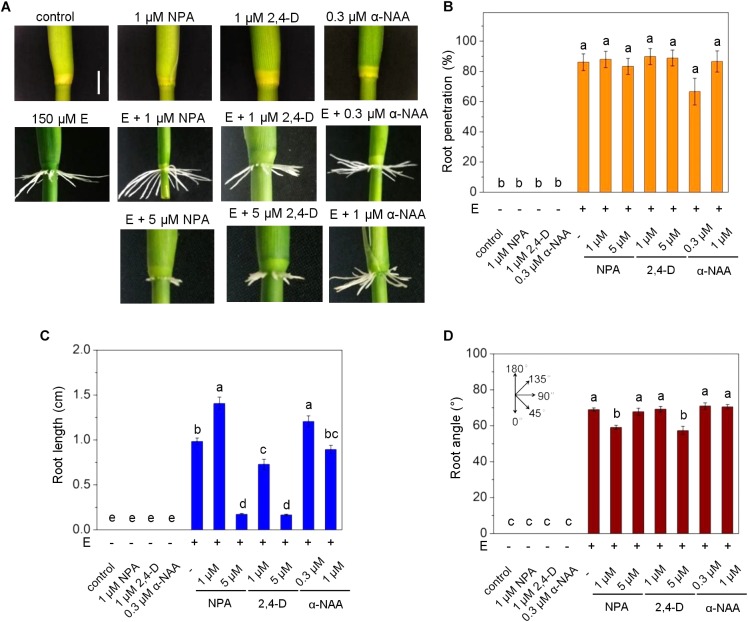
Auxin controls ethylene-induced AR elongation in the light. ARs were analyzed at the third node of stems treated with or without 150 ethephon and NPA, 2,4-D or α-NAA for 3 days in the light as indicated. **(A)** AR growth phenotypes (bar=1 cm). **(B)** Percentage of penetrated ARs. **(C)** Average lengths of penetrated ARs. **(D)** Average growth angle of ARs. Means (±SE) in **B–D** were determined from nine stems analyzed per treatment in three independent experiments. Different letters indicate statistically significant differences (*P* <0.05; ANOVA with Tukey test).

In order to investigate a possible cross-talk between auxin and ethylene in AR growth, we exposed stem sections to ethephon together with NPA, 2,4-D or α-NAA for 3 days (Figure 7). Neither 2,4-D or α-NAA nor auxin transport inhibition by NPA altered AR penetration in the light in the long term (Figure 7B) whereas root length was significantly elevated from 1 to 1.4 cm at 1 μM NPA and reduced to 0.2 cm at 5 μM NPA revealing a dose-dependent opposite response. Root elongation was also promoted by low (0.3 μM) α-NAA and was inhibited by 1 and 5 μM 2,4-D in a dose-dependent manner suggesting that the native auxin activity is at a suboptimal level in ethephon-induced ARs (Figure 7C). The growth angle was slightly reduced at 1 μM NPA and 5 μM 2,4-D and was unaffected by α-NAA (Figure 7D). These findings support the idea that ethylene overrides the inhibitory effect of NPA on AR emergence in the long run whereas control of AR elongation and, to a lesser degree, growth angle by auxin is not. Since NPA and 2,4-D reduced the growth angle in the dark and, to a minor degree, in the light, we next investigated a possible contribution of auxin in redirecting AR growth.

### Time Course Analysis of AR Redirection

**FIGURE 8:**
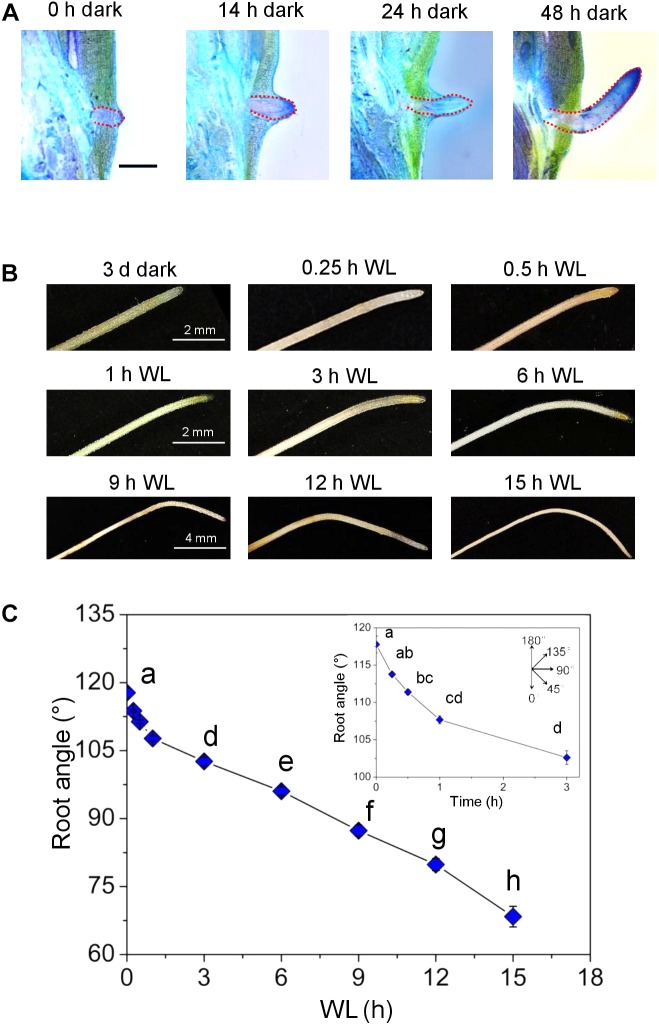
Light changes the growth angle within 30 min. **(A)** Stems sections including the third node were kept in the dark for up to 3 days. Longitudinal sections were obtained and stained with toluidine after 0, 14, 24, and 48 h to visualize AR growth (bar=2 mm). **(B)** Growth phenotype of ARs that were grown for 3 days in the dark and subsequently exposed to WL for the times indicated. **(C)** Changes in AR angle after exposure to light. Bars indicate means (±SE) from nine stem sections for per treatment analyzed in three independent experiments. Different letters indicate statistically significant differences (*P* < 0.05; ANOVA with Tukey test).

To study the mechanisms underlying redirection of AR growth, we established a time course of AR bending induced by light (Figure 8). Stem sections were exposed to darkness for 2 days to induce AR growth at an upward angle as visualized by toluidine-stained cross sections (Figure 8A). In the dark, emerged ARs were bending upward after 24 h. The upward angle of growth was fully established after 48 h. After 3 days in the dark, stems were exposed to white light (WL) and the root angle was monitored. Roots grew at an angle of 117.8° in the dark (Figure 8B,C). A significant change in growth direction to 111.4° was measured after 30 min. Within 15 h, the growth angle reached 68.3° which is about the angle at which ARs grow in the light (Figure 6D).

Gravitropic root growth is initiated by starch-containing statoliths in root cap statocytes. To exclude a possible role of statoliths in light-induced redirection of AR growth, statoliths were visualized at the root tip as brown precipitate from Lugols starch staining (Supplementary Figure S3). Neither exposure of stems to darkness in an upright or inverse orientation, nor a shift from dark to light for 1, 3 or 6 h altered the detectable pattern of starch staining.

### *OsPIN* Expression and Auxin Distribution in ARs That Change Their Growth Direction

To test whether a change in growth direction of ARs is accompanied by altered expression of *OsPIN* genes, we transferred rice stems that were kept in the dark for 3 days to induce AR growth at an upward angle, to the light for 0.5, 1, or 3 h (Supplementary Figure S2A). *OsPIN1b:GUS* expression was detected in the stele and in the root cap. Expression in the root cap was intensified within 3 h of exposure to light. A similar expression pattern was observed when ARs were exposed to ethephon in the light indicating that *OsPIN1b* expression is under light control (Supplementary Figure S2B). *OsPIN1c:GUS* was active at the root apex, xylem and stele tissues but not in lateral root cap cells. Neither transfer from dark to light (Supplementary Figure S2A) nor growth in the light with ethephon changed *OsPIN1c:GUS* expression (Supplementary Figure S2B). *OsPIN2:GUS* and *OsPIN10a:GUS* displayed a patchy expression pattern at AR tips in what appeared to be root cap cells that appeared to become stronger within 3 h after transfer to the light and weaker in the presence of NPA (Supplementary Figure S2C). Overall, the expression patterns were in accord with OsPIN1b and OsPIN1c-driven auxin transport in the stele, redistribution of auxin at the root tip and auxin transport in root cap cells by OsPIN2 and OsPIN10a. Altered expression of *OsPIN1b* and *OsPIN2* suggested an altered auxin distribution in response to the dark-to-light switch.

**FIGURE 9:**
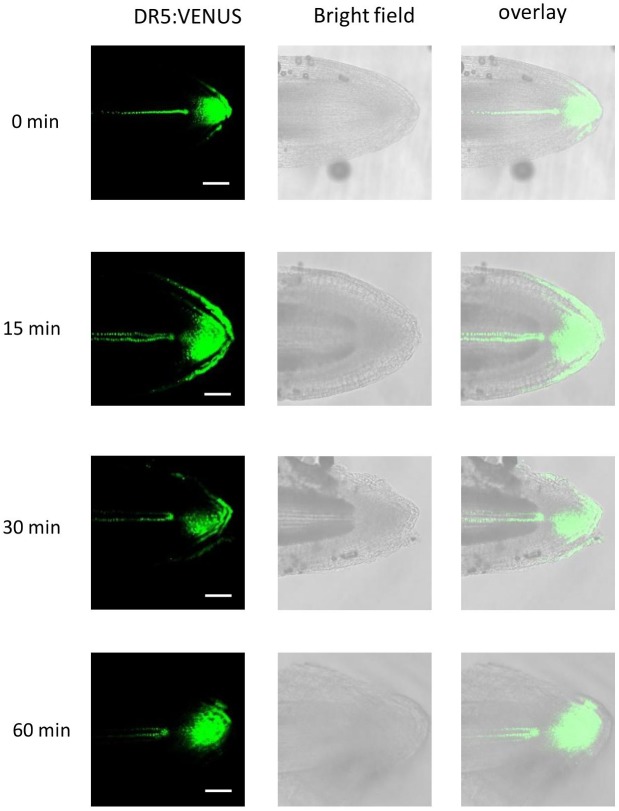
Light exposure triggers higher auxin activity at the lower side of AR tips. Stem section of the *DR5:VENUS* reporter line were kept in the dark for 5 days and subsequently transferred to the light for 15, 30, and 60 min. Auxin activity was detected by CLSM (bar=100 μm).

To test if altered *OsPIN* gene expression affects auxin distribution, we employed the DR5:VENUS reporter (Figure 9). AR growth was induced in the dark. After 5 days in the dark, the stems were transferred to the light and auxin distribution was visualized at the root tip for up to 1 h. Auxin appeared to accumulate at the lower side within 30 min suggesting that elevated auxin inhibits growth at this side resulting in downward bending of the root (Figure 8C). In summary, polar auxin transport likely regulates the angle at which ARs grow indicating that auxin plays a key role in shaping the AR system architecture in rice.

## Discussion

Plant root systems mainly consist of secondary roots that develop postembryonically from the primary root as lateral roots or from the shoot as ARs. Auxin is a key hormone that regulates secondary root formation and growth (Olatunji et al., 2017). In tomato, induction of ARs during flooding is mediated by ethylene that promotes auxin flux toward the submerged stem thereby increasing local auxin activity (Liu et al., 2009; Vidoz et al., 2010). In rice, the Lateral Organ Boundaries Domain (LBD) transcription factor Crown Rootless1/Adventitious Rootless1 (CRL1/ARL1) acts downstream of ARFs to control the formation of ARs (Inukai et al., 2005; Liu et al., 2005). Consequently, *arl1* mutants have fewer ARs at their stem nodes. While auxin is well known to promote formation of secondary roots including formation of ARs, the role of auxin in AR emergence and growth is less well studied. In rice, AR formation is developmentally programmed whereas emergence and growth are induced by environmental cues, rendering rice highly suitable to study these aspects. Flooding promotes AR emergence through ethylene signaling. The AR growth-promoting activity of ethylene is enhanced by gibberellic acid and repressed by abscisic acid (Steffens et al., 2006).

Our current study supported a role for polar auxin transport in shaping the AR system since inhibition of polar auxin transport by NPA reduced AR emergence, AR elongation growth and the angle at which roots grew in the dark. A similar phenotype was observed after treatment with the synthetic auxin 2,4-D. PIN proteins facilitate auxin efflux of the natural auxin IAA and of the synthetic auxin α-NAA but not of 2,4-D supporting the conclusion that high levels of 2,4-D accumulate in plant cells and thereby mimic inhibition of efflux carriers by NPA (Delbarre et al., 1996). By contrast, α-NAA did not similarly, alter AR growth, presumably because local auxin activity did not increase. Interestingly, when ARs were induced by ethylene to grow in the light, the growth rate was strongly repressed by NPA and 2,4-D whereas emergence and growth angle were not or only marginally affected suggesting that responsiveness to auxin differs in the light and in the dark. A previous study on rice showed that repression of the auxin efflux carrier gene *OsPIN1* repressed AR emergence supporting the conclusion that locally altered auxin activity controls AR development (Xu et al., 2005).

Analysis of defined *PIN* genes revealed differential expression patterns in ARs and epidermal cells above ARs. Expression patterns were in accord with their predicted functions based on protein similarity to Arabidopsis homologs. *PIN1b* and *PIN1c* were predominately expressed in the central cylinder as is *AtPIN1* which facilitates acropetal auxin movement. *PIN1b* and *PIN1c* were further expressed in the root cap where expression was enhanced when roots were exposed to light possibly indicating that *PIN1b* contributes to control of AR growth direction. Expression of *OsPIN1c* in AR primordia agrees with the observation that auxin levels are high at the primordia apex. High auxin activity at the root apex has also been shown for Arabidopsis (Petersson et al., 2009).

An unexpected and previously not reported finding was the accumulation of auxin specifically in epidermal cells covering root primordia but not in other epidermal cells. Programmed cell death (PCD) of epidermal cells was suggested to facilitate AR emergence (Mergemann and Sauter, 2000). Epidermal cells covering AR primordia were shown to have a unique molecular identity that allows them to undergo PCD in response to a specific trigger (Steffens and Sauter, 2009). Induction of PCD requires the hormonal signal ethylene and in addition a mechanical signal that provides spatial resolution and thereby limits PCD to the site where an AR emerges (Steffens et al., 2012). Ethylene signaling sets the stage in two ways, for one by promoting AR growth and, secondly, by activating the cell death program that, however, is executed only in cells that perceive mechanical force exerted by the growing AR primordium. Auxin that accumulates in epidermal cells could be synthesized locally in the epidermis itself or in the root tip from where it is transported to epidermal cells above it. However, auxin activity was low in AR primordia. Alternatively, efflux carrier-mediated auxin transport in epidermal cells could generate an auxin maximum in these cells. However, none of the efflux carrier genes analyzed was expressed at detectable levels in epidermal cells. Finally, it is possible, that local auxin synthesis is responsible for elevated auxin activity in these cells. The decline in auxin activity in epidermal cells above AR primordia upon growth induction of ARs in the dark occurs in parallel, and possibly as a consequence of, elevated *OsPIN2* expression in these epidermal cells and suggests a role of polar auxin transport in epidermal cells during AR emergence. Inhibition of polar auxin transport by NPA delayed epidermal cell death and reduced the rate of AR emergence. Taken together, our findings indicate that controlled local auxin activities through polar auxin transport contribute to rapid weakening of the epidermis as a physical barrier. At the same time, auxin activity in the root meristem increases likely promoting cell division activity.

The growth angle contributes substantially to AR system architecture. It is controlled by red and blue light signaling (Lin and Sauter, 2018). Upward AR growth in the dark is reverted to a downward angle in the light. The change in growth angle occurs within 30 min. Polar auxin transport is required to maintain an upward growth angle in the dark. It seems reasonable to assume that directed auxin flux is altered to change AR growth direction after exposure to light. Fluorescence auxin reporter data indicate that auxin accumulates at the lower side within 30 min. Auxin is known to inhibit root growth already at low concentrations. It is hence conceivable that growth is slowed down at this side resulting in downward bending of the AR tip. Whether differential expression of auxin carrier genes or regulation at the protein level alters efflux carrier activity remains to be resolved. In Arabidopsis, the growth direction of the primary root changes in response to hypoxic conditions such that the primary root no longer grows downward (Eysholdt-Derzso and Sauter, 2017). While auxin activity was asymmetrically distributed between epidermal cells of either side of the primary root, AtPIN2 protein, responsible for auxin transport in this cell layer, was symmetrically distributed. AtPIN2 distribution in the plasma membrane is regulated via phosphorylation/dephosphorylation (Santner and Watson, 2006; Michniewicz et al., 2007; Adamowski and Friml, 2015) pointing to multiple levels of auxin transport regulation in plants.

## Conclusion

Adventitious root emergence is controlled by polar auxin transport likely through activation of the auxin efflux carrier gene *OsPIN2* in epidermal cells above AR primordia and subsequent depletion of auxin in these cells. Polar auxin transport furthermore controls AR elongation and determines growth direction. Taken together, local auxin activities controlled by polar auxin transport through OsPIN efflux carriers is a major determinant of AR system architecture in rice.

## Materials and Methods

### Growth Conditions and Treatments

The seeds of the deepwater rice variety PG56 (*Oryza sativa* L., Pin Gaew 56) were originally obtained from the International Rice Research Institute (Philippines). The *OsPIN:GUS* lines in the rice *japonica* cultivar Nipponbare were generously donated by Prof. Chuanzao Mao (Zhejiang University, China). The *DR5:VENUS* line of the japonica rice cultivar 9522 was generously provided by Prof. Zheng Yuan (Shanghai Jiao Tong University, China). The VENUS protein is directed to the nucleus for better detection and resolution (Yang et al., 2017). Seeds were germinated in a 12.5 × 12.5 cm Petri dish with a surface-moist Whatman filter paper at 27°C in the dark for 4 days. Seedlings were transferred to 1.7-liter pots with a soil mixture consisting of two thirds of soil and one third of volcanic soil. Plants were grown in a growth chamber with 16 h light (362.3 μmol m^-2^s^-1^) at 27°C and 8 h dark at 19°C with 70% relative humidity for 12–14 weeks (Sauter, 1997). Stems were excised 2 cm below the third youngest node with a total length of 20 cm (Mergemann and Sauter, 2000). Stem sections were incubated in 150 ml beakers with 20 ml aqueous solution with or without different concentrations of ethephon, 2,4-D or α-NAA or NPA. Plastic cylinders covered the beakers to ensure high humidity. To investigate the effect of light on the growth angle of ARs, stem sections were either kept in the dark for 3 days to induce roots to emerge and subsequently exposed to WL (43 μmol m^-2^s^-1^) for the times indicated or exposed to light in the presence of the ethylene-releasing compound ethephon.

To determine the root angle, each AR was photographed (Canon SX 220 HS) and the angle measured with ImageJ (Fiji). Subsequently, roots were excised to determine the root length with a ruler.

### Evans Blue and Lugol Staining

Evans Blue staining was used for epidermal cell death determination and to better visualize AR penetration at the rice stem. Five mm of the node were cut and stained in a 2% (w/v) Evans Blue solution for 3 min followed by two washing steps in tap water. Root penetration was determined using a light microscope (Olympus SZ61, Japan) and the percentage was calculated based on the total number of AR initials that was taken as 100%. For Lugols staining, 1 cm of the root tip was isolated and stained with Lugols’ solution for 1 min. Subsequently, the samples were washed once in distilled water. The starch-containing statoliths were visualized and photographed using a binocular (Nikon H600L, Japan).

### Histochemical GUS Analysis

Histochemical β-glucuronidase (GUS) staining was performed according to Blázquez et al. (1997) with minor modifications. Rice stem sections were kept in the dark or exposed to WL for the duration indicated with or without 150 μM ethephon or 5 μM NPA. Isolated ARs or nodes were rapidly transferred to 90% pre-cooled acetone for 20 min and subsequently washed twice with 50 mM sodium-phosphate buffer (pH 7.2) for 5 min on a shaker. After removing the washing solution, 1 ml staining buffer (50 mM sodium phosphate, 0.2% [v/v] Triton X(-100, 2 mM K_4_[Fe(CN)_6_], 2 mM K_3_[Fe(CN)_6_], and 2 mM 5-bromo-4-chloro-3-indolyl-β-D-glucuronide [Duchefa], pH 7.5) was added to each sample. Samples were kept in a vacuum chamber for 10 min followed by shaking at 37°C in the dark (*OsPIN1b:GUS* and *OsPIN1c:GUS* for 1 h, *OsPIN2:GUS* and *OsPIN10a:GUS* overnight). The staining buffer was removed and samples were incubated in 70% ethanol to remove chlorophyll. GUS staining was visualized using a binocular (Nikon H600L, Japan).

### Confocal Laser Scanning Microscopy

Fluorescence images of *DR5:VENUS* samples were taken with a Leica SP5 confocal laser scanning microscope (CLSM). For an overview, a 5 mm segment containing an AR primordium and the surrounding nodal tissue was excised from the node. Root primordia and the epidermis were isolated and prepared on a slide with a drop of 10% (w/v) glycerol for imaging. Imaging of VENUS was done at 510–550 nm and bright field was regarded as a control. ARs that were transferred from dark to light were marked on the upper and lower sides to allow for proper orientation of the root in CLSM.

### Statistical Analysis

Statistical analysis was performed using Minitab. The *P*-value was set to *P* < 0.05. Comparison of means was analyzed for statistical difference with an analysis of variance (ANOVA) (Tukey test) or a two sample *t*-test.

## Author Contributions

CL and MS designed the project. CL performed the experiments. MS wrote the manuscript with contributions from CL.

## Conflict of Interest Statement

The authors declare that the research was conducted in the absence of any commercial or financial relationships that could be construed as a potential conflict of interest.
