# Supplementary material for: Polar Auxin Transport Determines Adventitious Root Emergence and Growth in Rice
Source: Front Plant Sci. 2019 Apr 9;10:444. doi: 10.3389/fpls.2019.00444 (PMC6465631; doi:10.3389/fpls.2019.00444)
Supplement: Supplementary file 1 [file Data_Sheet_1.PDF]

Supplementary Data

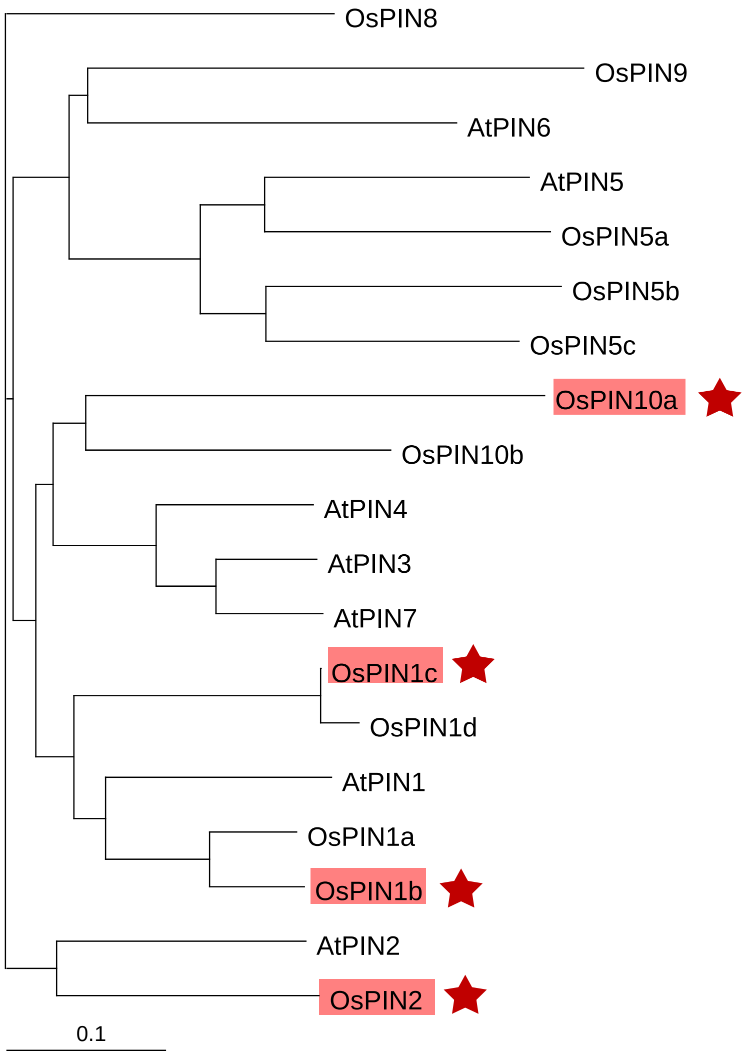

Supplementary Figure S1: Phylogenetic analysis of PIN family members in rice and arabidopsis. Multiple sequence alignment was performed with Clustal Omega and a phylogenetic tree was generated by Treeview; bar=0.1. Red stars indicate PIN genes that were analyzed with GUS reporter lines.

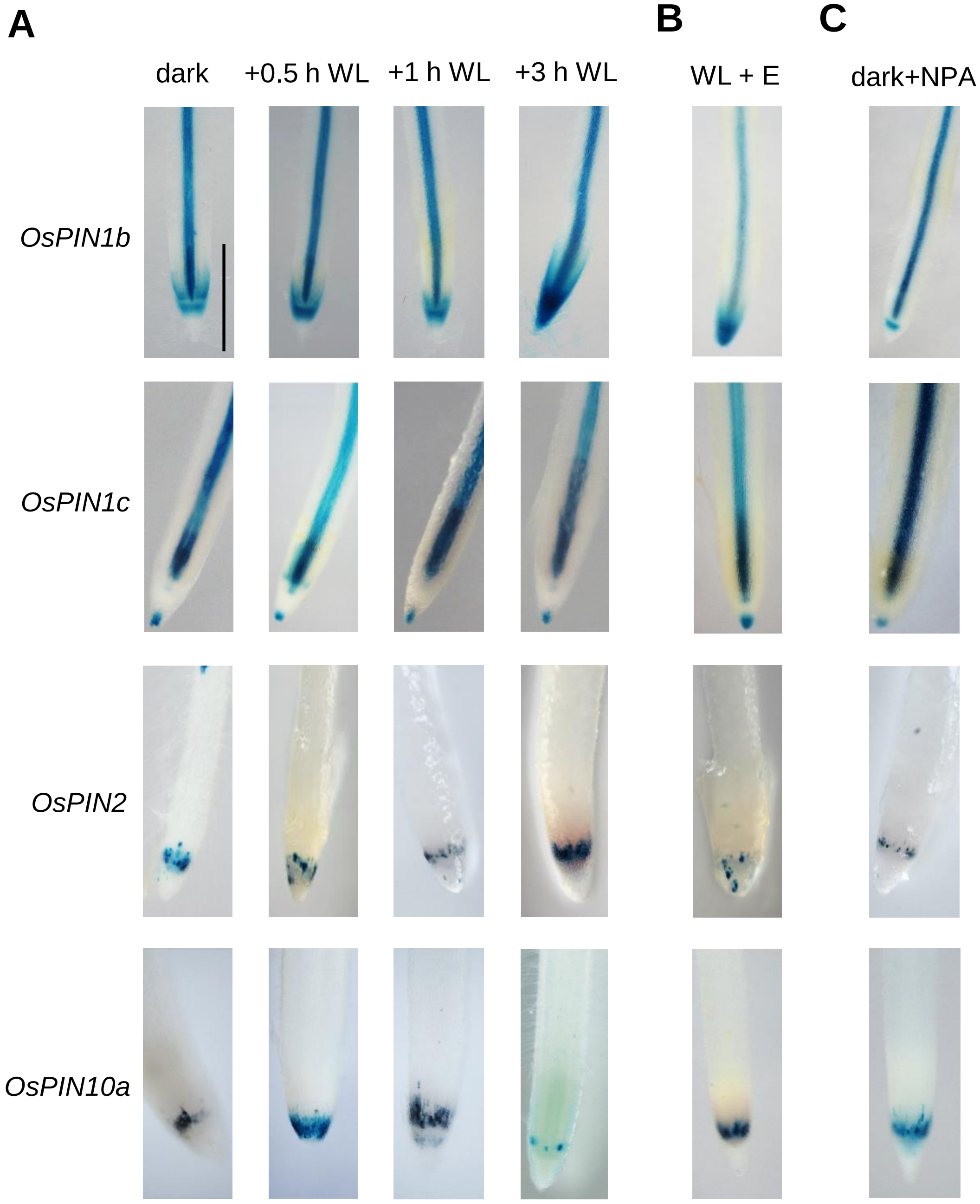

Supplementary Figure S2: Histochemical GUS analysis of *OsPIN* expression in rice ARs. Stem sections were kept in the dark for 3 d (dark), 3 d dark and 0.5 h white light (3 d Dark + 0.5 h WL), 3 d dark and 1 h white light (3 d Dark + 1 h WL), 3 d dark and 3 h white light (3 d Dark + 3 h WL), white light and 150 µM ethephon (WL + E) for 3 d, or in the dark with 5 µM NPA (dark + NPA). Images were obtained with a binocular (bar=0.5 mm).

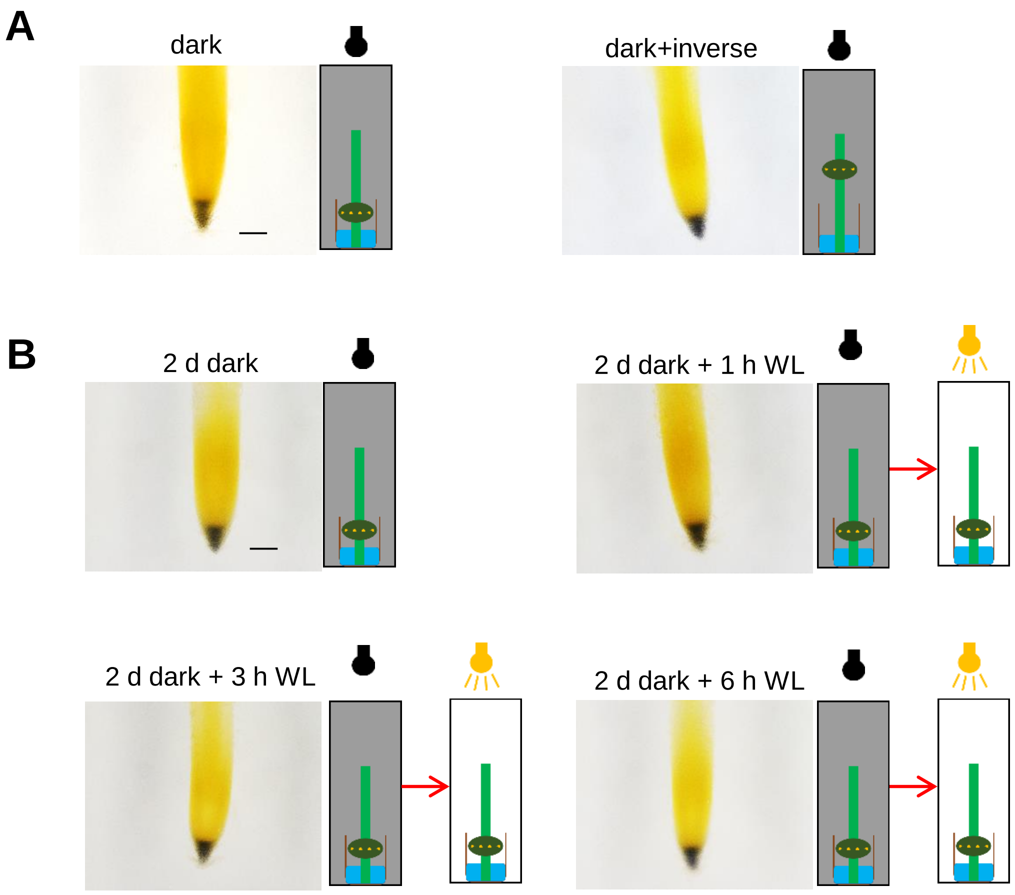

Supplementary Figure S3: Visualization of statoliths in AR caps.

(A) Stems were placed upright or in an inverse orientation in a beaker. After exposure to darkness for 2 d or 2 d darkness followed by 3 h light, ARs were isolated and stained with Lugols solution to visualize statolith-containing root cap cells (bar = 0.2 mm).

(B) Rice stems were kept in the dark for 2 d and subsequently exposed to light for 1 h, 3 h or 6 h. Isolated ARs were stained with Lugol solution as described in (A) (bar=0.2 mm).
